# Data on New Intermediate and Accidental Hosts Naturally Infected with *Angiostrongylus cantonensis* in La Gomera and Gran Canaria (Canary Islands, Spain)

**DOI:** 10.3390/ani13121969

**Published:** 2023-06-13

**Authors:** Natalia Martin-Carrillo, Edgar Baz-González, Katherine García-Livia, Virginia Amaro-Ramos, Néstor Abreu-Acosta, Jordi Miquel, Estefanía Abreu-Yanes, Román Pino-Vera, Carlos Feliu, Pilar Foronda

**Affiliations:** 1 Instituto Universitario de Enfermedades Tropicales y Salud Pública de Canarias (IUETSPC), Universidad de La Laguna (ULL), Avda. Astrofísico F. Sánchez, s/n, 38203 San Cristóbal de La Laguna, Canary Islands, Spain; nmartinc@ull.edu.es (N.M.-C.); ebazgonz@ull.edu.es (E.B.-G.); kgarcial@ull.edu.es (K.G.-L.); vamarora@ull.edu.es (V.A.-R.); gerencia@nertalab.es (N.A.-A.); esabya@gmail.com (E.A.-Y.); rpinover@ull.edu.es (R.P.-V.); 2 Department of Obstetricia y Ginecología, Pediatría, Medicina Preventiva y Salud Pública, Toxicología, Medicina Legal y Forense y Parasitología, Universidad de La Laguna (ULL), Avda. Astrofísico F. Sánchez, s/n, 38203 San Cristóbal de La Laguna, Canary Islands, Spain; 3 Programa de Doctorado Ciencias Médicas y Farmacéuticas, Desarrollo y Calidad de Vida, Universidad de La Laguna (ULL), Avda. Astrofísico F. Sánchez, s/n, 38203 San Cristóbal de La Laguna, Canary Islands, Spain; 4 Programa de Doctorado en Biodiversidad y Conservación, Universidad de La Laguna (ULL), Avda. Astrofísico F. Sánchez, s/n, 38203 San Cristóbal de La Laguna, Canary Islands, Spain; 5 Nertalab S.L. José Rodríguez Mouré, 4, Bajo, 38008 Santa Cruz de Tenerife, Canary Islands, Spain; 6 Departament de Biologia, Sanitat i Medi Ambient, Facultat de Farmàcia i Ciències de l’Alimentació, Universitat de Barcelona, Av. Joan XXIII, s/n, 08028 Barcelona, Ibérian Peninsula, Spain; jordimiquel@ub.edu (J.M.); cfeliu@ub.edu (C.F.); 7 Institut de Recerca de la Biodiversitat (IRBio), University of Barcelona, Av Diagonal, 645, 08028 Barcelona, Ibérian Peninsula, Spain

**Keywords:** *Angiostrongylus cantonensis*, eosinophilic meningitis, *Rattus rattus*, *Mus musculus*, *Felis catus*, *Limacus flavus*, *Milax gagates*, *Insulivitrina oromii*, *Insulivitrina emmersoni*

## Abstract

**Simple Summary:**

The rat lungworm, *Angiostrongylus cantonensis*, is the world’s leading cause of eosinophilic meningitis in humans. It is an emerging zoonotic parasite endemic to Asia and the Pacific Islands that has spread to all continents except Antarctica. In the Canary Islands, its presence was first detected in 2010 on the island of Tenerife. Numerous studies demonstrated the high capacity of *A. cantonensis* to colonize new areas, especially its ability to parasitize a wide range of animals. Due to the similarity of the ecosystems that we can find between the islands that make up the Canary Archipelago and the great diversity of species of both vertebrates and terrestrial gastropods, the objective of this study was to analyze several species as possible hosts of *A. cantonensis* on other islands in the Canary Islands, with the use of molecular tools. The present study confirmed the presence of *A. cantonensis* in two mammalian species, *Mus musculus* and *Felis catus*, and in four terrestrial gastropod species, *Limacus flavus, Milax gagates, Insulivitrina emmersoni*, and *Insulivitrina oromii*. The presence of *A. cantonensis* implies a possible risk to humans and other animals, which justifies the need for control measures to control the possible risk of infection and, thus, prevent public health and veterinary problems.

**Abstract:**

*Angiostrongylus cantonensis* is a metastrongyloid nematode and the etiologic agent of angiostrongyliasis, a disease characterized by eosinophilic meningitis. This emerging zoonotic parasite has undergone great expansion, including in some regions of Europe and America. In the Canary Islands, the parasite was first discovered parasitizing *Rattus rattus* on the island of Tenerife in 2010. To date, the distribution of this parasite in the Canary Islands has been restricted to the northern zone and the main cities of Tenerife. Using molecular tools for the sentinel species present in the Canary Islands, this study confirmed the presence of the nematode on two other islands in the Canary Archipelago: La Gomera and Gran Canaria. Furthermore, this emerging parasite was detected, besides in the common definitive host *R. rattus*, in wild *Mus musculus* and *Felis catus* and in four terrestrial gastropod species, *Limacus flavus, Milax gagates, Insulivitrina emmersoni*, and *Insulivitrina oromii*, two of them endemic to La Gomera, for the first time, increasing the number of non-definitive host species. This study reinforces the expansion character of *A. cantonensis* and highlights the importance of knowledge about sentinel species for identifying new transmission locations that help prevent and control the transmission of the parasite and, thus, prevent public health problems.

## 1. Introduction

The rat lungworm, *Angiostrongylus cantonensis*, is a parasitic nematode that causes a reemerging zoonotic disease, human eosinophilic meningitis, as well as neurological abnormalities in wild and domestic animals [1,2]. It has been reported worldwide since its first description in the Guangzhou region of China in the pulmonary arteries of brown rats, *Rattus norvegicus* [3]. This parasite has a complex life cycle involving various species of rats as definitive hosts [4], numerous gastropod species from widely different taxonomic groups as intermediate hosts, and various paratenic hosts. It was identified as a human pathogen in 1945 [5] and is now recognized as the leading cause of eosinophilic meningitis worldwide [6]. The most recent globally reported cases of human neuroangiostrongyliasis were approximately 7000 cases [7]. These data may be higher since many cases are not published. As non-permissive hosts, humans mainly become infected by eating raw or undercooked intermediate or paratenic hosts containing the infective larvae and by eating vegetables and salads contaminated with this parasite [7,8].

Upon ingestion, the infective larvae invade the intestinal wall, causing enteritis, and enter the bloodstream [9]. The central nervous system (CNS) is the most common site of migration and is the main clinical manifestation of eosinophilic meningitis. Human angiostrongyliasis presents with a broad clinical spectrum, ranging from mild disease to eosinophilic meningitis or encephalitis [10]. As a result, neurological damage and even death may occur, especially if prompt and proper treatment is not administered [11,12,13].

Traditionally, *A. cantonensis* was an endemic parasite of the tropical and subtropical areas of the Far East, including Southeast Asia, the Pacific Islands, areas of South and Central America, and the Caribbean [9,14]. It was widely spread, mainly across the tropics and subtropics [15]. More recently, it was reported in rodents (*Rattus rattus*) on the Atlantic archipelago of the Canary Islands (Spain) [16]; on the Mediterranean island of Mallorca (Spain) parasitizing hedgehog, *Atelerix algirus* [17]; and later in the rodent populations (*R. rattus* and *R. norvegicus*) of continental Europe (Valencia, Spain) [18]. These data suggest that despite the lack of tolerance of *A. cantonensis*, which has traditionally been largely limited to tropical and subtropical regions, to cold temperatures [19], with the fulcrum of its presence in Europe, the parasite could spread across the continent to more temperate regions [15], as already mentioned in Australia [20] and the United States [21]. These data suggest the geographic expansion of the parasite, resulting in a possible rapid increase in the incidence of infection in humans [22].

The influence of increasing factors, such as globalization and climate change, may influence the invasion of non-endemic regions by lungworms. Furthermore, many authors attributed the spread of *A. cantonensis* to the great diversity of its intermediate hosts and its high adaptability to new intermediate host species [21]. In addition, the invasion of *A. cantonensis* was associated with cargo shipments by aircraft or ships, which sometimes unintentionally import rats and gastropods [9,23]. 

The Canary Islands constitute an archipelago consisting of eight volcanic islands located in the Atlantic Ocean that settled on the African plate. This archipelago is in the Northwest of the African Coast near Southern Morocco and northern Western Sahara (13°23–18°8 W and 27°37–29°24 N). It is one of the outermost regions of the European Union and a part of the natural region of Macaronesia, of which it is the largest and most populated archipelago. The population of the Canary Archipelago is mainly concentrated on its two capital islands, Tenerife and Gran Canaria, at approximately 43% and 40%, respectively [24]. In the Canary Islands, *A. cantonensis* was detected in *R. rattus* on the island of Tenerife in 2010 [16] and posteriorly in three mollusk species [25] and in the endemic lizards *Gallotia galloti* [26]. Although previous studies were carried out with rodents from all the Canary Islands, *A. cantonensis* was only detected in Tenerife [23]. 

Taking into account the rapid dispersal of *A. cantonensis* worldwide and the health implications for humans and wildlife, there is a need to determine the distribution of *A. cantonensis* in the Canary Archipelago. Given the similarity of the climatic and orographic conditions between Tenerife and the other islands of the Canary Archipelago and the fact that *A. cantonensis* was reported in non-definitive hosts, such as *A. algirus*, in other regions [17], the aim of this study was to detect the presence of *A. cantonensis* using molecular tools in different possible host species on two other Canary Islands, La Gomera and Gran Canaria. 

## 2. Materials and Methods

### 2.1. Samples Collection

**Figure 1:**
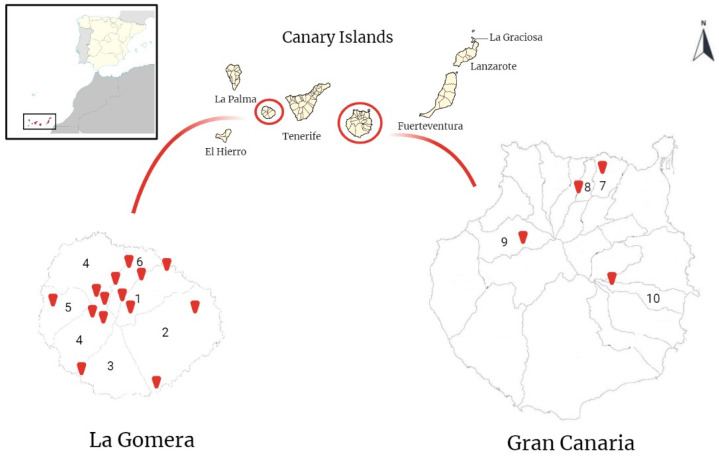
Map of the sampled areas on the islands of La Gomera and Gran Canaria (Canary Islands, Spain). In the upper left corner, the geographical situation of the Canary Islands is shown in relation to the African continent and the Iberian Peninsula. The red marks show the sampled locations. The numbers indicate the sampled municipalities. 1: Hermigua; 2: San Sebastian de La Gomera; 3: Alajeró; 4: Vallehermoso; 5: Valle Gran Rey; 6: Agulo; 7: Arucas; 8: Firgas; 9: Artenara; 10: Ingenio. (Image created with BioRender.com; accessed on 30 March 2023).

From February 2022 to January 2023, sampling campaigns were conducted in rural and urban areas on La Gomera and Gran Canaria islands (Figure 1). On the island of La Gomera, rural areas are mainly composed of Laurisilva and Fayal-Brezal zones, biotopes with the presence of horizontal rain biotopes. On Gran Canaria, rural areas are composed of cultivated fields, ravines, and pine forests.

Samples of rodents of *Mus musculus* (60), *R. norvegicus* (4), and *R. rattus* (40) species were obtained from six and four municipalities of La Gomera and Gran Canaria islands, respectively (Figure 1 and Table 1 and Table 2), using live traps. Traps were set in the afternoon and collected the following day in the morning. Once captured, euthanasia was performed with CO_2_ or by cervical dislocation for rats and mice, respectively. Animal work was approved and authorized by “Consejería de Transición Ecológica, Lucha contra el Cambio Climático y Planificación Territorial” (Gobierno de Canarias) (Expte. 2021/51732).

**Figure 2:**
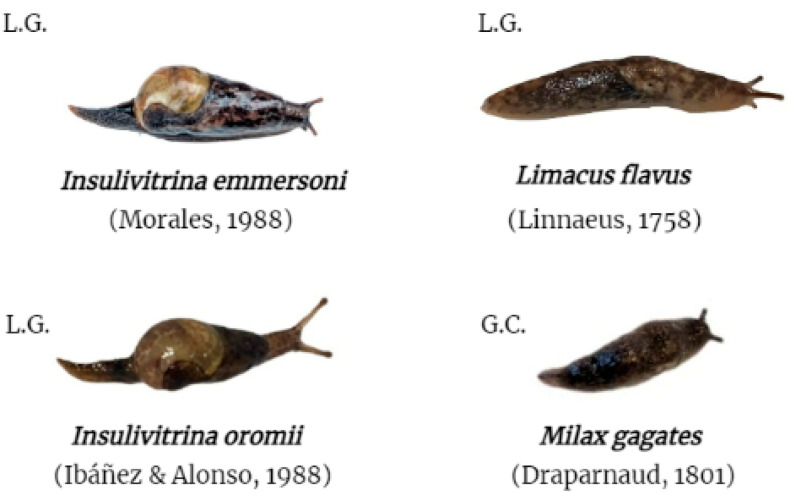
Terrestrial gastropod species included in the study. L.G. collected on the island of La Gomera; G.C. collected on the island of Gran Canaria.

Further, terrestrial gastropods were collected from five municipalities in La Gomera and one in Gran Canaria (Figure 2 and Table 1 and Table 2). The collected terrestrial gastropods included the endemic species *Insulivitrina emmersoni* (28) and *Insulivitrina oromii* (8), present only on the island of La Gomera (Figure 2 and Table 1 and Table 2). On the other hand, *Limacus flavus* (10) and *Milax gagates* (24), possible native species to the Canary Islands, were collected on the islands of La Gomera and Gran Canaria, respectively (Figure 2 and Table 1 and Table 2). This field study was authorized by the “Consejería de Transición Ecológica, Lucha contra el Cambio Climático y Planificación Territorial” (Gobierno de Canarias) (Expte. 19-2022/1110092705). The capture of animals in protected areas (Garajonay National Park) was approved by “Cabildo Insular de La Gomera” (Expte. 4121/2022) and “Parque Nacional de Garajonay” (749.153, TELP/122.918).

On the other hand, *Felis catus* (40) samples from La Gomera were also analyzed (Table 1 and Table 2). These animals were donated for this study by Gobierno de Canarias staff (approved by the Gobierno de Canarias, expedient number 2022/25073, and Excmo. Cabildo Insular de La Gomera, expedient numbers 1872/2021 and 1821/2022).

### 2.2. Search for Angiostrongylus cantonensis and Sample Isolation

After dissection of rodents, the lungs and hearts were isolated in physiological saline and examined with a Leica M80 stereo microscope (Leica Mikrosysteme Vertrieb GmbH, Wetzlar, Germany) to detect the presence of *A. cantonensis*. For terrestrial gastropods, tissue samples were placed in 1 mL of 0.01% pepsin0.7% HCl for digestion and observed under the Leica DM750 Microscopium model ICC50 HD (Leica Microsystems, Heerbrugg, Switzerland) for the search for larval forms of *A. cantonensis*.

On the other hand, for rodents and *F. catus*, approximately 1 g of the liver was aseptically removed using a sterile scalpel. For terrestrial gastropods, samples were cut from the posterior end of the mollusk foot (~0.5 g) under aseptic conditions. The samples were fixed in absolute ethanol and stored at 4 °C until DNA extraction. 

### 2.3. DNA Extraction

Genomic DNA was extracted using approximately 25 mg of liver tissue, and the posterior end of terrestrial gastropod foot tissue was cut into small pieces and used for DNA extraction. Other tissues were not included in the analyses, following the success of Anettová et al. [26] looking for DNA of *A. cantonensis* in the livers of paratenic hosts. The tissues were deposited in tubes containing a mixture of 250 µL of a lysis solution composed of 30 mM Tris-HCL pH 8.0 mM EDTA and 0.4% SDS, and three microliters of proteinase K (20 mL^−1^) (PanReac AppliChem ITW Reagents, Germany) was added and incubated at 56 °C overnight. Then, 250 µL of NH_4_Ac 4M was added, thoroughly mixed, and subsequently incubated for 30 min at room temperature (15–25 °C). The mix was spun for 10 min at 13,000 rpm, and the pellet was discarded. DNA was then precipitated from the supernatant with ethanol, and the pellet was suspended in 200 µL of 1X TE (10 mM Tris-HCL pH 8.1 mM EDTA) [27]. The quantity and quality of genomic DNA were checked using DeNovix DS-11 + Spectrophotometer (DeNovix Inc., Wilmington, DE, USA) (Table S1).

### 2.4. PCR Amplification

For the detection of *A. cantonensis* in the extracted genomic DNA, nested PCR was performed using the primers (10 µM) described by Qvarnstrom et al. [28] to amplify the entire region of internal spacer 1 (ITS1) and the second PCR with specific primers for *A. cantonensis*, as described by Izquierdo-Rodríguez et al. [29]. Both rounds of PCR contained 1X buffer (VWR International, Haasrode, Belgium), 0.2 mM of each dNTP (VWR International, Haasrode, Belgium), 1.5 mM MgCl_2_ (VWR International, Haasrode, Belgium), and 20–40 ng of total genomic DNA for each sample in a total volume of 25 µL. Positive controls (gDNA extracted from *A. cantonensis* adult worm with the procedure described by López et al. [27]) and negative controls (H_2_O) were included in all assays to verify nested PCR performance. All DNA samples were run in duplicate. Amplification was performed with the XP Cycler (Bioer Technology, Hangzhou, China) using the following parameters: 94 °C for 5 min; 35 cycles at 94 °C for 30 s, 57 °C for 90 s, and 72 °C for 90 s; and a final extension at 72 °C for 10 min. The resulting amplifications were checked on 1.5% agarose gel. 

### 2.5. Sequencing and Sequencing Data Analysis

PCR products with the expected size (642-bp) were sequenced at Macrogen Spain (Madrid, Spain) with primers ITS1 Canto F3 and ITS1 Canto R1 [29]. The sequences obtained using the Sanger method were interpreted with MEGA X software [30], using the multiple alignment program ClustalW included in MEGA X, and minor corrections were made by hand. Subsequently, the sequences were analyzed using the Basic Local Alignment Search Tool (BLAST), and their identities were confirmed by homology comparison.

Phylogenetic relationships based on the neighbor joining [31] method were carried out with the Kimura 2-parameter model [32], and 1000 bootstrap replications explored the relationships among them. The sequence of *Protostrongylus oryctolagi* (Acc. Number: OM307447) was used as the outgroup. 

## 3. Results

After observation of the rodent lungs and hearts, the nematode *A. cantonensis* was not observed in any of the specimens. In the case of terrestrial gastropods, microscopic observation was negative for the presence of *A. cantonensis* larvae. However, it should be noted that some of the individuals had observed structures similar to the larvae of this parasite, but due to the poor conditions in which they were found, it was impossible to perform a morphometric study of them. Regarding the molecular study, *A. cantonensis* was detected on the two analyzed islands, La Gomera and Gran Canaria, and in all the analyzed species, both vertebrates and invertebrates. 

The overall prevalence of *A. cantonesis* was 15% in *M. musculus* (9/60); 12.5% (5/40) in feral cats, and 27.14% in terrestrial gastropods (*I. oromii*: 37.5% (3/8); *I. emmersoni*: 32.14% (9/28); *L. flavus*: 60% (6/10); and *M. gagates*: 4.16% (1/24)). The prevalence data for all the analyzed hosts and areas are shown in Table 2.

The molecular results for the definitive hosts revealed the presence of *A. cantonensis* in the 2.5% (1/40) of *R. rattus* that were analyzed, concretely in the municipality of Hermigua (7.14%; 1/14) on La Gomera island.

The BLAST analysis confirmed that the sequences obtained from all the hosts analyzed in this study showed 100% homology with *A. cantonensis* (Query Cover (Q.C.): 100%). With respect to the other Angiostrongylidae species, the BLAST analyses showed a lower identity: 84.8% with *Angiostrongylus vasorum* (Q.C.: 39%) and *Angiostrongylus chabaudi* (Q.C.: 40%) and 81% with *Angiostrongylus abstrusus* (Q.C.: 19%).

**Figure 3:**
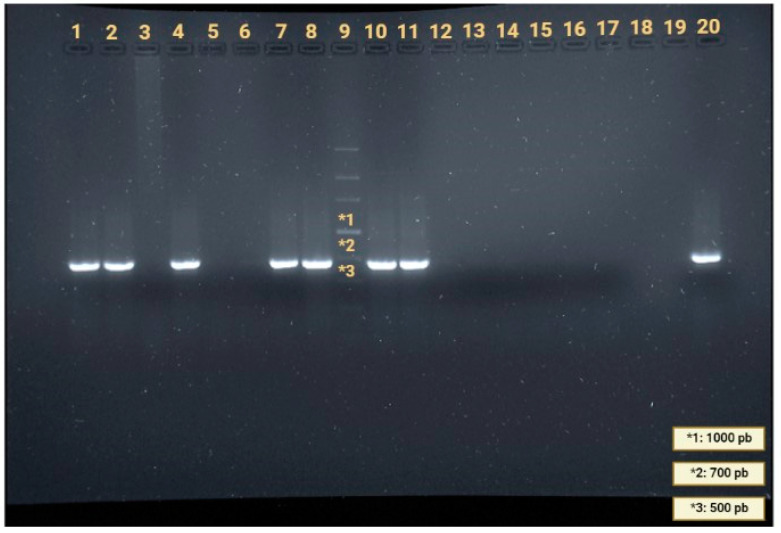
Results of nested PCR for *Angiostrongylus cantonensis* (642 bp) in samples included in this study. Line 1: *Felis catus*; Lane 2: *Mus musculus*; Line 4: *Rattus rattus*; Line 7: *Insulivitrina emmersoni*; Line 8: *Insulivitrina oromii*; Line 10: *Limacus flavus*; Line 11: Milex gagates; Line 19: negative control; Line 20: positive control; Lines 3, 5, 6, and 12–18: negative samples for *A. cantonensis* DNA; Line 9: molecular ladder ranging from 100 to 4000 bp (VWR International, Haasrode, Belgium). (1*, 2*, and 3* each refer to the above lines.)

Of the 34 amplified samples obtained by nested PCR (Figure 3), 16 sequences were obtained for the 18 S ribosomal RNA gene partial sequence, the internal transcribed spacer 1 complete sequence, and the 5.8S ribosomal RNA gene partial sequence. Phylogenetic analyses confirmed the identity of *A. cantonensis* observed by BLAST with an average Query Cover and Identity of 99.8% and 99%, respectively.

**Figure 4:**
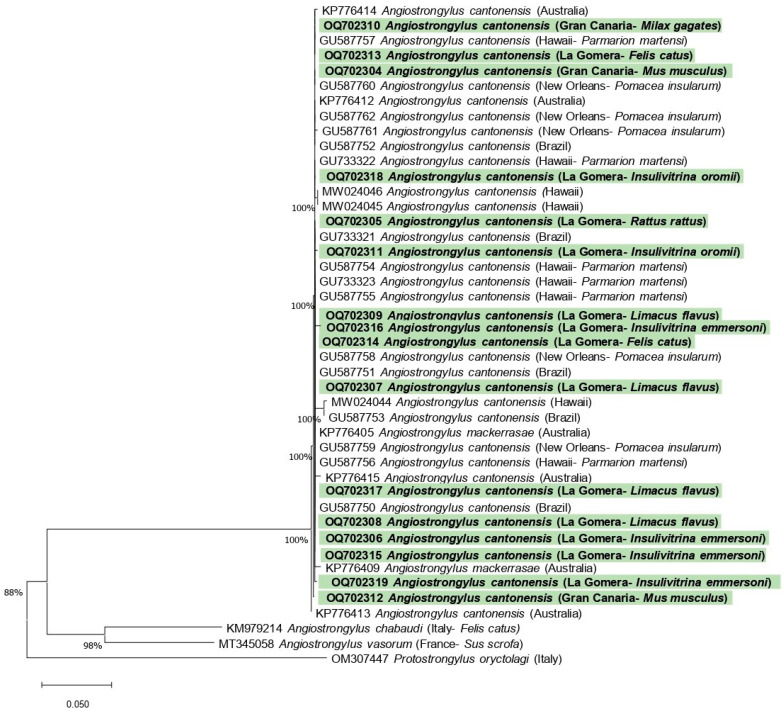
Phylogenetic relationships between sequences of the partial sequence of the internal transcribed spacer 1 of *Angiostrongylus* spp., including the nucleotide sequences obtained in this study (shown in bold and highlighted in green). The tree was built using neighbor joining method based on genetic distance calculated by the Kimura 2-parameter model. There were a total of 482 positions in the final data set.

The nucleotide sequences obtained in this study were submitted to the GenBank database under accession numbers OQ702304–OQ702319. An alignment of 482 bp was used for phylogenetic analysis. The results of the neighbor joining analysis based on the partial sequence of internal transcribed spacer 1 are shown in Figure 4.

## 4. Discussion

The present study constitutes the first detection of *A. cantonensis* on the islands of La Gomera and Gran Canaria (Canary Islands, Spain). Previous studies reported this nematode on the neighboring island of Tenerife, but it was not detected on any other Canary Islands [16,23]. Therefore, the present results demonstrate the expansion of *A. cantonensis* to new locations in the Canary Archipelago, confirming the colonizer character of this nematode, recently widely reported by other authors, such as Barrat et al. [22], and Cowie et al. [15], among others. In the present work, on La Gomera and Gran Canaria, *A. cantonensis* was detected by molecular methods both in mammals and invertebrates; specifically, in two of the analyzed rodent species, *M. musculus* and *R. rattus*; in *F. catus*; and in the four analyzed terrestrial gastropod species, *I. emmersoni*, *I. oromii*, *L. flavus*, and *M. gagates*; all of these are the first reports, except for *R. rattus*, of natural infection in these host species. 

The role of many rodent species as definitive hosts of *A. cantonensis* is widely known [22]. These mammals are present in different ecosystems that are widely distributed throughout the world. In this study, we included the species *M. musculus* and *F. catus* on the list of accidental hosts, which are also present worldwide. Both species were introduced to the Canary Islands and are present on the eight islands that make up the Canary Archipelago. 

Despite the numerous studies carried out on *A. cantonensis* in *M. musculus*, it was only reported in experimentally infected mice [33,34]. With the detection of genetic material of *A. cantonensis* in wild *M. musculus*, we can confirm the ability of this vertebrate to be naturally infected by *A. cantonensis*, increasing the list of the accidental hosts of this parasite. This host may also play a fundamental role in the dispersion to non-endemic regions.

Regarding *F. catus*, there are no previous data about its parasitization by *A. cantonensis*. In the Canary Islands, the feral cat is widely distributed on all the islands, being a top predator in the vertebrate food chain [35]. Its role as an accidental host of *A. cantonensis* is now confirmed with the detection of its DNA. This fact makes sense considering that its diet includes mammals, birds, reptiles, gastropods, and arthropods [36,37], many of which were confirmed as hosts of *A. cantonensis* in the Canary Islands, as is the case of the endemic lizard *G. galloti*, recently cited as a paratenic host [26]. It is important to note that the presence of *A. cantonensis* in feral cats does not involve health risks for humans.

Regarding the matter of the studied gastropod species, the four analyzed species presented DNA of *A. cantonensis*, also increasing the number of known intermediate hosts. *Limacus flavus* and *M. gagates* are common species with a wide distribution in the Canary Islands and are also widely distributed in Europe, Asia, and South America [38]. However, *I. oromii* and *I. emmersoni* are endemic to the Canary Islands and are exclusively found on the island of La Gomera in the biotopes of Laurisilva and Monteverde [38]. This finding is consistent with the previously demonstrated ability of *A. cantonensis* to naturally infect a wide variety of freshwater and terrestrial gastropods species [25,39]. 

The biotopes where *A. cantonensis* was detected coincides with the biotopes where this parasite had previously been detected on the island of Tenerife [23]. Specifically, on the island of La Gomera, the areas where it is located coincide with the areas of incidence of trade winds and horizontal rain, and on the island of Gran Canaria the areas where it is located coincide with the urban and peri-urban areas.

Some studies relate the rate of development of *A. cantonensis* in intermediate hosts or sentinel species to climate [40]. The survival of many of the hosts of this parasite depends on optimal humidity and temperature conditions for their development and life cycle, as can be the case for many terrestrial gastropods. Overall, in most natural systems, climate change affects hosts and parasites by altering their survival, reproduction, and transmission, among other factors [21,41,42]. Additionally, increased international trade, travel to disease-endemic regions, and changes in dietary habits are collectively expected to facilitate the further range expansion of *A. cantonensis* [43,44]. This fact was verified by numerous studies citing this emerging zoonotic parasite in many regions of the world under diverse environmental conditions [15,21].

The current rapid expansion of a multitude of emerging pathogens indicates that the control levels of many of them must be increased to avoid public health problems, both locally and globally. Many studies advocate the use of sentinel animals because of their potential [45], and, by studying a wide range of potential hosts, we can increase vigilance regarding the spread of emerging pathogens such as *A. cantonensis*.

Autochthonous or introduced species that act as hosts in regions recently invaded by *A. cantonensis* were previously identified [41,45,46], as in this study. Hence, further research is needed to confirm the presence of the rat lungworm in a wide range of animals that can act as sentinel species and even as intermediate or paratenic hosts. One factor that should be considered for any future epidemiological studies of this emerging pathogen is the technique that we used to detect its presence, since, as we were able to verify in this study, it is not always possible to visualize the adult in definitive hosts. Therefore, the use of more sensitive techniques is vital to rule out false negatives in species with the potential to harbor the parasite.

In many regions, awareness of the rat lungworm is low, not only among the general community but also among physicians and health authorities. Therefore, the education of medical professionals and local and tourist populations must be prioritized in endemic areas. This makes even more sense if we observe that the spread of this emerging zoonotic pathogen has exponentially increased over the years.

## 5. Conclusions

Overall, the results of this study indicate, for the first time, the presence of *A. cantonensis* on La Gomera and Gran Canaria, in the Canary Islands, using molecular tools. In addition, the parasite was first reported in naturally infected *M. musculus* and *F. catus* and in four species of terrestrial gastropods: *I. emmersoni, I. oromomii, L. flavus*, and *M. gagates*. All of these have a wide distribution, except for the gastropod species *I. emmersoni* and *I. oromii*, which are endemic to La Gomera. Based on these data, this study reinforces the high potential for the expansion of *A. cantonensis* and stresses the importance of the knowledge that sentinel species have when controlling infectious or parasitic diseases to indicate possible sources of risk of infection.

## Figures and Tables

**Table 1:** Origin of analyzed host species in this study.

Host Species	Island	Sample Size	Total
*Felis catus*	La Gomera	40	40
*Mus musculus*	La Gomera	34	60
	Gran Canaria	26	
*Rattus norvegicus*	La Gomera	4	4
*Rattus rattus*	La Gomera	33	40
	Gran Canaria	7	
*Insulivitrina oromii*	La Gomera	8	8
*Insulivitrina emmersoni*	La Gomera	28	28
*Limacus flavus*	La Gomera	10	10
*Milax gagates*	Gran Canaria	24	24
		Total	214

**Table 2:** Prevalence of *Angiostrongylus cantonensis* obtained on the La Gomera and Gran Canaria islands (Canary Islands, Spain) in this study, divided by host species as well as location.

Island	Location	Host Species	Prevalence of *Angiostrongylus cantonensis* P (%) * (+/n)	Total P (%) * (+/n)
La Gomera	Agulo	*Felis catus*	0 (0/2)	11.11 (1/9)
		*Mus musculus*	0 (0/5)	
		*Insulivitrina oromii*	50 (1/2)	
	Alajeró	*Felis catus*	0 (0/1)	0 (0/6)
		*Mus musculus*	0 (0/3)	
		*Insulivitrina oromii*	0 (0/2)	
	Hermigua	*Felis catus*	12.5 (1/8)	25% (9/36)
		*Mus muculus*	0 (0/15)	
		*Insulivitrina oromii*	66.06 (2/3)	
		*Limacus flavus*	60 (6/10)	
	San Sebastián de la Gomera	*Mus musculus*	0 (0/6)	0 (0/6)
	Valle Gran Rey	*Felis catus*	0 (0/6)	0 (0/7)
		*Insulivitrina oromii*	0 (0/1)	
	Vallehermoso	*Felis catus*	17.39 (4/23)	23.21 (13/56)
		*Mus musculus*	0 (0/5)	
		*Insulivitrina emmersoni*	32.14 (9/28)	
Total of La Gomera				19.16 (23/120)
Gran Canaria	Artenara	*Mus musculus*	0 (0/2)	0 (0/2)
	Arucas	*Mus musculus*	16.66 (1/6)	16.66 (1/6)
	Firgas	*Mus musculus*	100 (1/1)	8 (2/25)
		*Milax gagates*	4.16 (1/24)	
	Ingenio	*Mus musculus*	41.17 (7/17)	41.17 (7/17)
Total of Gran Canaria				20 (10/50)
Total				13.52% (33/170)

* Prevalence of *Angiostrongylus cantonensis* %; (+/n): positive samples for nested PCR/analyzed samples.

## Data Availability

Not applicable.

## Supplementary Materials

The following supporting information can be downloaded at: https://www.mdpi.com/article/10.3390/ani13121969/s1, Table S1: Quantification of the DNA obtained in the study samples.
