# Supplementary material for: Data on New Intermediate and Accidental Hosts Naturally Infected with Angiostrongylus cantonensis in La Gomera and Gran Canaria (Canary Islands, Spain)
Source: Animals (Basel). 2023 Jun 13;13(12):1969. doi: 10.3390/ani13121969 (PMC10295084; doi:10.3390/ani13121969)
Supplement: Supplementary file 1 [file animals-13-01969-s001.zip › animals-2427477-supplementary.pdf]

Table S1. Quantification of the DNA obtained in the study samples.

Sample code	Host specie	Island	Municipality	ng/µl ADN	Dilution used	Result Nested-PCR	Sequences Acc. Number
FcH1	*Felis catus*	La Gomera	Vallehermoso	10.28	-	Negative	-
FcH2	*Felis catus*	La Gomera	Vallehermoso	16.36	-	Negative	-
FcH3	*Felis catus*	La Gomera	Valle Gran Rey	1534.38	1:80	Negative	-
FcH4	*Felis catus*	La Gomera	Vallehermoso	71.99	1:5	Negative	-
FcH5	*Felis catus*	La Gomera	Alajeró	675.61	1:50	Negative	-
FcH6	*Felis catus*	La Gomera	Vallehermoso	39.62	-	Negative	-
FcH7	*Felis catus*	La Gomera	Hermigua	1414.88	1:80	Negative	-
FcH8	*Felis catus*	La Gomera	Vallehermoso	9.89	-	Negative	-
FcH9	*Felis catus*	La Gomera	Vallehermoso	59.57	1:5	**Positive**	*
FcH10	*Felis catus*	La Gomera	Vallehermoso	7.63	-	**Positive**	OQ702313
FcH11	*Felis catus*	La Gomera	Vallehermoso	15.76	-	Negative	-
FcH12	*Felis catus*	La Gomera	Vallehermoso	9.88	-	**Positive**	*
FcH13	*Felis catus*	La Gomera	Vallehermoso	26.77	-	Negative	-
FcH14	*Felis catus*	La Gomera	Vallehermoso	8.15	-	Negative	-
FcH15	*Felis catus*	La Gomera	Vallehermoso	39.55	1:5	**Positive**	*
FcH16	*Felis catus*	La Gomera	Vallehermoso	9.75	-	Negative	-
FcH17	*Felis catus*	La Gomera	Vallehermoso	3.45	-	Negative	-
FcH18	*Felis catus*	La Gomera	Vallehermoso	72.51	1:10	**Positive**	OQ702314
FcH19	*Felis catus*	La Gomera	Hermigua	6.61	-	Negative	-
FcH20	*Felis catus*	La Gomera	Vallehermoso	287.53	1:30	Negative	-
FcH21	*Felis catus*	La Gomera	Valle Gran Rey	253.87	1:25	Negative	-

Sample code	Host specie	Island	Municipality	ng/µl ADN	Dilution used	Result Nested-PCR	Sequences Acc. Number
FcH22	*Felis catus*	La Gomera	Valle Gran Rey	565.88	1:50	Negative	-
FcH23	*Felis catus*	La Gomera	Valle Gran Rey	49.45	-	Negative	-
FcH24	*Felis catus*	La Gomera	Vallehermoso	64.20	1:5	Negative	-
FcH25	*Felis catus*	La Gomera	Vallehermoso	1401.63	1:95	Negative	-
FcH26	*Felis catus*	La Gomera	Agulo	538	1:40	Negative	-
FcH27	*Felis catus*	La Gomera	Vallehermoso	683	1:50	Negative	-
FcH28	*Felis catus*	La Gomera	Agulo	883	1:70	Negative	-
FcH29	*Felis catus*	La Gomera	Vallehermoso	717	1:65	Negative	-
FcH30	*Felis catus*	La Gomera	Vallehermoso	865	1:60	Negative	-
FcH31	*Felis catus*	La Gomera	Vallehermoso	398.52	1:30	Negative	-
FcH32	*Felis catus*	La Gomera	Vallehermoso	1169.89	1:100	Negative	-
FcH33	*Felis catus*	La Gomera	Hermigua	650.04	1:60	Negative	-
FcH34	*Felis catus*	La Gomera	Vallehermoso	219.78	1:20	Negative	-
FcH35	*Felis catus*	La Gomera	Hermigua	318.27	1:30	Negative	-
FcH36	*Felis catus*	La Gomera	Hermigua	503.86	1:50	Negative	-
FcH37	*Felis catus*	La Gomera	Valle Gran Rey	25.59	1:20	Negative	-
FcH38	*Felis catus*	La Gomera	Hermigua	336.52	1:30	Negative	-
FcH39	*Felis catus*	La Gomera	Hermigua	206.58	1:20	Negative	-
FcH40	*Felis catus*	La Gomera	Hermigua	596.64	1:50	Negative	-
RH1	*Rattus rattus*	La Gomera	Hermigua	289.73	1:30	Negative	-
RH2	*Rattus rattus*	La Gomera	Hermigua	1860	1:200	Negative	-
RH3	*Rattus rattus*	La Gomera	Hermigua	1705	1:179	Negative	-
RH4	*Mus musculus*	La Gomera	Hermigua	3207	1:309	Negative	-

Sample code	Host specie	Island	Municipality	ng/µl ADN	Dilution used	Result Nested-PCR	Sequences Acc. Number
RH5	*Mus musculus*	La Gomera	Hermigua	533	1:50	Negative	-
RH6	*Mus musculus*	La Gomera	Hermigua	489	1:50	Negative	-
RH7	*Rattus rattus*	La Gomera	Hermigua	574	1:50	Negative	-
RH8	*Rattus rattus*	La Gomera	San Sebastián de L.G.	1179	1:110	Negative	-
RH9	*Mus musculus*	La Gomera	Alajeró	1550	1:120	Negative	-
RH10	*Mus musculus*	La Gomera	Alajeró	2310	1:180	Negative	-
RH11	*Mus musculus*	La Gomera	Alajeró	3821	1:300	Negative	-
RH12	*Rattus rattus*	La Gomera	Alajeró	1673	1:17	Negative	-
RH13	*Rattus norvegicus*	La Gomera	Hermigua	511	1:50	Negative	-
RH14	*Rattus rattus*	La Gomera	Hermigua	667	1:50	Negative	-
RH15	*Rattus norvegicus*	La Gomera	Hermigua	342	1:40	Negative	-
RH16	*Rattus rattus*	La Gomera	Hermigua	1400	1:100	Negative	-
RH17	*Rattus norvegicus*	La Gomera	Hermigua	6.76	-	Negative	-
RH18	*Mus musculus*	La Gomera	Hermigua	784	1:60	Negative	-
RH19	*Mus musculus*	La Gomera	Hermigua	667	1:70	Negative	-
RH20	*Mus musculus*	La Gomera	Hermigua	297	1:30	Negative	-
RH21	*Mus musculus*	La Gomera	Hermigua	290	1:30	Negative	-
RH22	*Mus musculus*	Gran Canaria	Ingenio	3252	1:310	Negative	-
RH23	*Mus musculus*	Gran Canaria	Ingenio	1271	1:130	Negative	-
RH24	*Mus musculus*	Gran Canaria	Ingenio	4720	1:410	**Positive**	OQ702304
RH25	*Mus musculus*	Gran Canaria	Ingenio	1627	1:120	Negative	-
RH26	*Mus musculus*	Gran Canaria	Ingenio	2617	1:120	Negative	-
RH27	*Mus musculus*	Gran Canaria	Ingenio	5140	1:510	Negative	-

**Sample code**	**Host specie**	**Island**	**Municipality**	**ng/µl ADN**	**Dilution used**	**Result Nested-PCR**	**Sequences Acc. Number**
RH28	*Mus musculus*	Gran Canaria	Ingenio	6756	1:610	Negative	-
RH29	*Rattus rattus*	Gran Canaria	Ingenio	4156	1:410	Negative	-
RH30	*Mus musculus*	Gran Canaria	Artenara	2779	1:210	Negative	-
RH31	*Mus musculus*	Gran Canaria	Artenara	226	1:210	Negative	-
RH32	*Mus musculus*	Gran Canaria	Ingenio	800	1:50	Negative	-
RH33	*Mus musculus*	Gran Canaria	Ingenio	1027	1:100	**Positive**	OQ702312
RH34	*Mus musculus*	Gran Canaria	Ingenio	1261	1:200	Negative	-
RH35	*Mus musculus*	Gran Canaria	Ingenio	2468	1:210	Negative	-
RH36	*Mus musculus*	Gran Canaria	Ingenio	2871	1:250	**Positive**	*
RH37	*Rattus rattus*	Gran Canaria	Ingenio	4334	1:410	Negative	-
RH38	*Mus musculus*	Gran Canaria	Arucas	2725	1:210	Negative	-
RH39	*Mus musculus*	Gran Canaria	Arucas	377	1:110	Negative	-
RH40	*Mus musculus*	Gran Canaria	Arucas	587	1:50	Negative	-
RH41	*Mus musculus*	Gran Canaria	Arucas	604	1:60	Negative	-
RH42	*Mus musculus*	Gran Canaria	Arucas	1265	1:200	Negative	-
RH43	*Rattus rattus*	Gran Canaria	Arucas	2219	1:210	Negative	-
RH44	*Mus musculus*	La Gomera	Vallehermoso	4720	1:450	Negative	-
RH45	*Mus musculus*	La Gomera	Vallehermoso	907	1:90	Negative	-
RH46	*Rattus rattus*	La Gomera	Vallehermoso	2281	1:130	Negative	-
RH47	*Rattus norvegicus*	La Gomera	Vallehermoso	1544	1:149	Negative	-
RH48	*Rattus rattus*	La Gomera	Vallehermoso	5277	1:500	Negative	-
RH49	*Rattus rattus*	La Gomera	Vallehermoso	1677	1:150	Negative	-
RH50	*Mus musculus*	La Gomera	Vallehermoso	3219	1:130	Negative	-

Sample code	Host specie	Island	Municipality	ng/µl ADN	Dilution used	Result Nested-PCR	Sequences Acc. Number
RH51	*Mus musculus*	La Gomera	Vallehermoso	1607	1:150	Negative	-
RH52	*Mus musculus*	La Gomera	Vallehermoso	2234	1:210	Negative	-
RH53	*Rattus rattus*	La Gomera	Vallehermoso	2338	1:210	Negative	-
RH54	*Rattus rattus*	La Gomera	Vallehermoso	668	1:60	Negative	-
RH55	*Mus musculus*	Gran Canaria	Ingenio	2016.20	1:120	**Positive**	*
RH56	*Mus musculus*	Gran Canaria	Ingenio	768.41	1:70	**Positive**	*
RH57	*Mus musculus*	Gran Canaria	Ingenio	1013.16	1:100	**Positive**	*
RH58	*Mus musculus*	Gran Canaria	Ingenio	875.47	1:80	Negative	-
RH59	*Mus musculus*	Gran Canaria	Ingenio	234.75	1:120	**Positive**	*
RH60	*Rattus rattus*	Gran Canaria	Ingenio	873.18	1:80	Negative	-
RH61	*Rattus rattus*	Gran Canaria	Ingenio	1691.03	1:130	Negative	-
RH62	*Rattus rattus*	Gran Canaria	Ingenio	1022.66	1:100	Negative	-
RH63	*Rattus rattus*	Gran Canaria	Ingenio	3855.67	1:300	Negative	-
RH64	*Mus musculus*	Gran Canaria	Firgas	1525.76	1:110	**Positive**	*
RH65	*Mus musculus*	Gran Canaria	Arucas	2629.43	1:120	**Positive**	*
RH66	*Rattus rattus*	La Gomera	Hermigua	1696.12	1:100	Negative	-
RH67	*Rattus rattus*	La Gomera	Hermigua	919.52	1:40	**Positive**	OQ702305
RH68	*Rattus rattus*	La Gomera	Valle Gran Rey	1163.28	1:60	Negative	-
RH69	*Rattus rattus*	La Gomera	Valle Gran Rey	571.44	1:20	Negative	-
RH70	*Rattus rattus*	La Gomera	Valle Gran Rey	402.40	1:20	Negative	-
RH71	*Rattus rattus*	La Gomera	Valle Gran Rey	506.54	1:25	Negative	-
RH72	*Rattus rattus*	La Gomera	Valle Gran Rey	1097.19	1:50	Negative	-
RH73	*Rattus rattus*	La Gomera	Hermigua	737.24	1:30	Negative	-

Sample code	Host specie	Island	Municipality	ng/µl ADN	Dilution used	Result Nested-PCR	Sequences Acc. Number
RH74	*Rattus rattus*	La Gomera	Hermigua	68.11	-	Negative	-
RH75	*Rattus rattus*	La Gomera	San Sebastián de L.G.	10793.87	1:50	Negative	-
RH76	*Rattus rattus*	La Gomera	San Sebastián de L.G.	574.38	1:30	Negative	-
RH77	*Rattus rattus*	La Gomera	San Sebastián de L.G.	407.48	1:20	Negative	-
RH78	*Mus musculus*	La Gomera	San Sebastián de L.G.	2024.63	1:100	Negative	-
RH79	*Mus musculus*	La Gomera	San Sebastián de L.G.	1545.60	1:80	Negative	-
RH80	*Rattus rattus*	La Gomera	Agulo	1198.36	1:50	Negative	-
RH81	*Rattus rattus*	La Gomera	Agulo	330.02	1:15	Negative	-
RH82	*Mus musculus*	La Gomera	Agulo	1100.57	1:50	Negative	-
RH83	*Mus musculus*	La Gomera	Agulo	482.28	1:20	Negative	-
RH84	*Mus musculus*	La Gomera	Agulo	377.03	1:15	Negative	-
RH85	*Rattus rattus*	La Gomera	Hermigua	2169.72	1:100	Negative	-
RH86	*Rattus rattus*	La Gomera	Hermigua	114.43	1:5	Negative	-
RH87	*Mus musculus*	La Gomera	Hermigua	1510.14	1:80	Negative	-
RH88	*Mus musculus*	La Gomera	Hermigua	761.98	1:40	Negative	-
RH89	*Mus musculus*	La Gomera	Hermigua	716.46	1:30	Negative	-
RH90	*Mus musculus*	La Gomera	Hermigua	2032.36	1:90	Negative	-
RH91	*Mus musculus*	La Gomera	Hermigua	235.38	1:10	Negative	-
RH92	*Mus musculus*	La Gomera	San Sebastián de L.G.	2637.99	1:100	Negative	-
RH93	*Mus musculus*	La Gomera	San Sebastián de L.G.	1295.93	1:60	Negative	-
RH94	*Mus musculus*	La Gomera	San Sebastián de L.G.	253.07	1:10	Negative	-
RH95	*Mus musculus*	La Gomera	San Sebastián de L.G.	1380.43	1:60	Negative	-
RH96	*Ratus rattus*	La Gomera	San Sebastián de L.G.	1456.06	1:70	Negative	-

Sample code	Host specie	Island	Municipality	ng/µl ADN	Dilution used	Result Nested-PCR	Sequences Acc. Number
RH97	*Mus musculus*	La Gomera	Hermigua	335.13	1:15	Negative	-
RH98	*Mus musculus*	La Gomera	Hermigua	6918.97	1:300	Negative	-
RH99	*Mus musculus*	La Gomera	Hermigua	77.25	-	Negative	-
RH100	*Rattus rattus*	La Gomera	Hermigua	210.36	1:10	Negative	-
RH101	*Rattus rattus*	La Gomera	Hermigua	265.81	1:20	Negative	-
RH102	*Mus musculus*	La Gomera	Agulo	1165.44	1:50	Negative	-
RH103	*Rattus rattus*	La Gomera	Agulo	2409.57	1:100	Negative	-
RH104	*Mus musculus*	La Gomera	Agulo	20.53	1:70	Negative	-
BAB1-01	*Insulivitrina emmersoni*	La Gomera	Vallehermoso	775	1:70	Negative	-
BAB1-02	*Insulivitrina emmersoni*	La Gomera	Vallehermoso	1325	1:110	Negative	-
BAB1-03	*Insulivitrina emmersoni*	La Gomera	Vallehermoso	1481	1:120	Negative	-
BAB1-04	*Insulivitrina emmersoni*	La Gomera	Vallehermoso	1696	1:170	**Positive**	*
BAB1-05	*Insulivitrina emmersoni*	La Gomera	Vallehermoso	1056	1:100	Negative	-
BAB1-06	*Insulivitrina emmersoni*	La Gomera	Vallehermoso	1007	1:00	Negative	-
BAB1-07	*Insulivitrina emmersoni*	La Gomera	Vallehermoso	1243	1:100	**Positive**	OQ702315
BAB1-08	*Insulivitrina emmersoni*	La Gomera	Vallehermoso	1370	1:130	Negative	-
BAB1-09	*Insulivitrina emmersoni*	La Gomera	Vallehermoso	885	1:80	Negative	-
BAB1-10	*Insulivitrina emmersoni*	La Gomera	Vallehermoso	1352	1:130	**Positive**	OQ702306
BAB1-11	*Insulivitrina emmersoni*	La Gomera	Vallehermoso	1492.86	1:110	Negative	-
BAB2-01	*Insulivitrina emmersoni*	La Gomera	Vallehermoso	574.15	1:40	Negative	-
BAB2-02	*Insulivitrina emmersoni*	La Gomera	Vallehermoso	445.53	1:20	Negative	-
BAB2-03	*Insulivitrina emmersoni*	La Gomera	Vallehermoso	921.30	1:50	Negative	-
BAB2-04	*Insulivitrina emmersoni*	La Gomera	Vallehermoso	1314.62	1:60	**Positive**	OQ702316

Sample code	Host specie	Island	Municipality	ng/µl AND	Dilution used	Result Nested-PCR	Sequences Acc. Number
BAB2-05	*Insulivitrina emmersoni*	La Gomera	Vallehermoso	603.02	1:30	Negative	-
BAB2-06	*Insulivitrina emmersoni*	La Gomera	Vallehermoso	1534.86	1:90	Negative	-
BAB2-07	*Insulivitrina emmersoni*	La Gomera	Vallehermoso	948.03	1:50	Negative	-
BAB3-01	*Insulivitrina emmersoni*	La Gomera	Vallehermoso	1921.14	1:150	**Positive**	*
BAB3-02	*Insulivitrina emmersoni*	La Gomera	Vallehermoso	1616.40	1:130	Negative	-
BAB3-03	*Insulivitrina emmersoni*	La Gomera	Vallehermoso	1808.68	1:140	**Positive**	*
BAB3-04	*Insulivitrina emmersoni*	La Gomera	Vallehermoso	1175.73	1:110	Negative	-
BAB3-05	*Insulivitrina emmersoni*	La Gomera	Vallehermoso	1959.59	1:150	**Positive**	*
BAB3-06	*Insulivitrina emmersoni*	La Gomera	Vallehermoso	2006.29	1:200	Negative	-
BAB3-07	*Insulivitrina emmersoni*	La Gomera	Vallehermoso	433.57	1:50	**Positive**	*
BAB3-08	*Insulivitrina emmersoni*	La Gomera	Vallehermoso	1066.50	1:100	**Positive**	*
BAB3-09	*Insulivitrina emmersoni*	La Gomera	Vallehermoso	675.72	1:60	Negative	-
BAB3-10	*Insulivitrina emmersoni*	La Gomera	Vallehermoso	850.84	1:80	Negative	-
BAB4-01	*Insulivitrina emmersoni*	La Gomera	Vallehermoso	1943.92	1:100	Negative	-
BAB4-02	*Insulivitrina emmersoni*	La Gomera	Vallehermoso	1624.91	1:90	Negative	-
GCG-01	*Milax gagates*	Gran Canaria	Firgas	393.20	1:30	Negative	-
GCG-02	*Milax gagates*	Gran Canaria	Firgas	627.82	1:60	Negative	-
GCG-03	*Milax gagates*	Gran Canaria	Firgas	3096.30	1:300	Negative	-
GCG-04	*Milax gagates*	Gran Canaria	Firgas	1121.08	1:100	Negative	-
GCG-05	*Milax gagates*	Gran Canaria	Firgas	555.01	1:50	Negative	-
GCG-06	*Milax gagates*	Gran Canaria	Firgas	1592.41	1:150	**Positive**	*
GCG-07	*Milax gagates*	Gran Canaria	Firgas	406.00	1:40	Negative	-
GCG-08	*Milax gagates*	Gran Canaria	Firgas	1451.76	1:120	Negative	-

Sample code	Host specie	Island	Municipality	ng/µl AND	Dilution used	Result Nested-PCR	Sequences Acc. Number
GCG-09	*Milax gagates*	Gran Canaria	Firgas	959.94	1:90	Negative	-
GCG-10	*Milax gagates*	Gran Canaria	Firgas	186.69	1:10	Negative	-
GCP-01	*Milax gagates*	Gran Canaria	Firgas	494.01	1:40	Negative	-
GCP-02	*Milax gagates*	Gran Canaria	Firgas	392.13	1:30	Negative	-
GCP-03	*Milax gagates*	Gran Canaria	Firgas	503.46	1:50	Negative	-
GCP-04	*Milax gagates*	Gran Canaria	Firgas	496.58	1:40	Negative	-
GCP-05	*Milax gagates*	Gran Canaria	Firgas	293.31	1:20	Negative	-
GCP-06	*Milax gagates*	Gran Canaria	Firgas	650.12	1:60	**Positive**	OQ702310
GCP-07	*Milax gagates*	Gran Canaria	Firgas	268.44	1:20	Negative	-
GCP-08	*Milax gagates*	Gran Canaria	Firgas	206.02	1:20	Negative	-
GCP-09	*Milax gagates*	Gran Canaria	Firgas	179.40	1:10	Negative	-
GCP-10	*Milax gagates*	Gran Canaria	Firgas	283.42	1:20	Negative	-
GCR-01	*Milax gagates*	Gran Canaria	Firgas	270.01	1:20	Negative	-
GCR-02	*Milax gagates*	Gran Canaria	Firgas	829.61	1:80	Negative	-
GCR-03	*Milax gagates*	Gran Canaria	Firgas	996.80	1:90	Negative	-
GCR-04	*Milax gagates*	Gran Canaria	Firgas	1222.08	1:110	Negative	-
LG-Fla-01	*Limacus flavus*	La Gomera	Hermigua	4685	1:220	**Positive**	*
LG-Fla-02	*Limacus flavus*	La Gomera	Hermigua	2334	1:100	Negative	-
LG-Fla-03	*Limacus flavus*	La Gomera	Hermigua	834	1:40	**Positive**	OQ702307
LG-Fla-04	*Limacus flavus*	La Gomera	Hermigua	519	1:25	Negative	-
LG-Fla-05	*Limacus flavus*	La Gomera	Hermigua	638	1:30	**Positive**	*
LG-Fla-06	*Limacus flavus*	La Gomera	Hermigua	2673	1:120	**Positive**	OQ702308
LG-Fla-07	*Limacus flavus*	La Gomera	Hermigua	1278	1:5	**Positive**	OQ702309

Sample code	Host specie	Island	Municipality	ng/µl ADN	Dilution used	Result Nested-PCR	Sequences Acc. Number
LG-Fla-08	*Limacus flavus*	La Gomera	Hermigua	901	1:40	**Positive**	OQ702317
LG-Fla-09	*Limacus flavus*	La Gomera	Hermigua	3107	1:120	Negative	-
LG-Fla-10	*Limacus flavus*	La Gomera	Hermigua	446	1:20	Negative	-
LG-Orom-01	*Insulivitrina oromii*	La Gomera	Hermigua	282	1:10	**Positive**	OQ702311
LG-Orom-02	*Insulivitrina oromii*	La Gomera	Hermigua	1630	1:80	Negative	-
LG-Orom-03	*Insulivitrina oromii*	La Gomera	Hermigua	537	1:20	**Positive**	OQ702318
LG-Orom-04	*Insulivitrina oromii*	La Gomera	Agulo	444	1:20	**Positive**	OQ702319
LG-Orom-05	*Insulivitrina oromii*	La Gomera	Agulo	1041	1:50	Negative	-
LG-Orom-06	*Insulivitrina oromii*	La Gomera	Alajeró	140	-	Negative	-
LG-Orom-07	*Insulivitrina oromii*	La Gomera	Alajeró	361	1:20	Negative	-
LG-Orom-08	*Insulivitrina oromii*	La Gomera	Valle Gran Rey	289	1:10	Negative	-

* The sequences obtained were not of sufficient quality for phylogenetic analyses.
